# Multigene Panel Sequencing Reveals Cancer-Specific and Common Somatic Mutations in Colorectal Cancer Patients: An Egyptian Experience

**DOI:** 10.3390/cimb44030090

**Published:** 2022-03-18

**Authors:** Amira Salah El-Din Youssef, Mohamed A. Abdel-Fattah, Mai M. Lotfy, Auhood Nassar, Mohamed Abouelhoda, Ahmed O. Touny, Zeinab K. Hassan, Mohammed Mohey Eldin, Abeer A. Bahnassy, Hussein Khaled, Abdel Rahman N. Zekri

**Affiliations:** 1 Cancer Biology Department, National Cancer Institute, Cairo University, Cairo 11796, Egypt; mai.lotfy@nci.cu.edu.eg (M.M.L.); auhood.nassar@nci.cu.edu.eg (A.N.); zeinab.hassan@nci.cu.edu.eg (Z.K.H.); 2 Biotechnology Department, Faculty of Agriculture, Ain-Shams University, Cairo 11566, Egypt; m_abdelfatah@agr.asu.edu.eg; 3 Faculty of Engineering, Cairo University, Cairo 12613, Egypt; mabouelhoda@gmail.com; 4 Surgical Oncology Department, National Cancer Institute, Cairo University, Cairo 11796, Egypt; ahmed.touny@nci.cu.edu.eg; 5 Tropical Medicine Department, El Kasr Al-Aini, Cairo University, Cairo 11562, Egypt; m.mohy@cu.edu.eg; 6 Molecular Pathology Department, National Cancer Institute, Cairo University, Cairo 11796, Egypt; chaya2000@hotmail.com; 7 Medical Oncology Department, National Cancer Institute, Cairo University, Cairo 11796, Egypt; khussein528@gmail.com

**Keywords:** multigene sequencing, colorectal cancer, Egyptian, somatic mutations

## Abstract

This study aims at identifying common pathogenic somatic mutations at different stages of colorectal carcinogenesis in Egyptian patients. Our cohort included colonoscopic biopsies collected from 120 patients: 20 biopsies from patients with inflammatory bowel disease, 38 from colonic polyp patients, and 62 from patients with colorectal cancer. On top of this, the cohort included 20 biopsies from patients with non-specific mild to moderated colitis. Targeted DNA sequencing using a customized gene panel of 96 colorectal related genes running on the Ion Torrent NGS technology was used to process the samples. Our results revealed that 69% of all cases harbored at least one somatic mutation. Fifty-seven genes were found to carry 232 somatic non-synonymous variants. The most frequently pathogenic somatic mutations were localized in *TP53*, *APC*, *KRAS*, and *PIK3CA.* In total, 16 somatic mutations were detected in the CRC group and in either the IBD or CP group. In addition, our data showed that 51% of total somatic variants were CRC-specific variants. The average number of CRC-specific variants per sample is 2.4. The top genes carrying CRC-specific mutations are *APC*, *TP53*, *PIK3CA*, *FBXW7*, *ATM*, and *SMAD4.* It seems obvious that *TP53* and *APC* genes were the most affected genes with somatic mutations in all groups. Of interest, 85% and 28% of the *APC* and *TP53* deleterious somatic mutations were located in Exon 14 and Exon 3, respectively. Besides, 37% and 28% of the total somatic mutations identified in *APC* and *TP53* were CRC-specific variants, respectively. Moreover, we identified that, in 29 somatic mutations in 21 genes, their association with CRC patients was unprecedented. Ten detected variants were likely to be novel: six in *PIK3CA* and four variants in *FBXW7*. The detected P53, Wnt/βcatenin, Angiogenesis, EGFR, TGF-β and Interleukin signaling pathways were the most altered pathways in 22%, 16%, 12%, 10%, 9% and 9% of the CRC patients, respectively. These results would contribute to a better understanding of the colorectal cancer and in introducing personalized therapies for Egyptian CRC patients.

## 1. Introduction

Colorectal cancer (CRC) is one of the leading causes of mortality and morbidity worldwide. It is the third most common neoplasm and the second leading cause of cancer-related death worldwide [1]. In Egypt, CRC was ranked seventh among the most common malignant tumors with around 3000 cases, representing 4% of totally diagnosed cancers and 53% of gastrointestinal tract (GIT) cancers [2,3]. In 2020, CRC was still ranked among the top 10, with 3430 new cases in that year (https://gco.iarc.fr (accessed on 15 June 2021). Its rank jumped in females, however, to the fifth place (56% females and 44% males against 48% females and 52% males in 2015) [2,3]. It is estimated that around 75% of the CRC cases arise sporadically through a cascade of acquired somatic genomic alterations, while 5–10% are of hereditary origin [4]. The pathogenesis of the CRC is very heterogeneous and influenced by multiple factors related to dietary habits, genetic predisposition, long-standing inflammatory bowel disease and presence of colorectal polyps [4].

Next generation sequencing (NGS) technology is an efficient means to characterize mutations associated with the disease in the patients’ genomes [5]. It is fast and cost effective, enabling the study of variants in affected patients at different disease stages and in different tissues. This provides more in-depth insights into the mutational processes functioning in various types of cancers, which eventually enhances our understanding regarding the biology of the disease and, accordingly, leads to better patient management and genetic screening [6]. Targeted sequencing is a modality of NGS technology to sequence a set of genes of interest. Compared to whole genome and whole exome sequencing, this method has the advantage of reducing the cost per sample, increasing the depth, and running multiple samples at the same time [7]. The increased depth of target sequencing has the extra advantage, even over targeted PCR-based technique, of detecting somatic variants at very low allele frequencies [8].

A number of studies have been published to characterize the somatic mutations and related genes associated with CRC worldwide [4,5,6,7]. In this research, we attempted to answer the question of whether Egyptian patients had a comparable pattern of somatic changes to those observed in other countries. We used a gene panel of colorectal-related genes to determine the landscape of the somatic mutations in a cohort of 140 samples from Egyptian patients: 20 biopsy samples with mild to moderate colitis and 120 colonoscopic biopsy samples from patients at different stages of colorectal carcinogenesis. As shown in the results section, this study could indeed identify the most relevant genes and frequent mutations in the Egyptian populations, which is expected to contribute to more accurate diagnostics and better disease management.

## 2. Materials and Methods

### 2.1. Patient Samples

Fresh colonoscopic biopsy samples (*n* = 120) were collected from the patients classified into (1) inflammatory bowel disease (IBD; *n* = 20), (2) colonic polyp (CP; *n* = 38) and (3) colorectal carcinoma patients (CRC; *n* = 62) as well as extra participants with chronic non-specific mild to moderate colitis without any colonoscopic abnormalities (Colitis; *n* = 20). The collected colonoscopic biopsies were stored in MACS Tissue Storage Solution at −80° freezer until DNA extraction. Before the study began, each patient who was enrolled signed a written informed consent form. The Institutional Review Board of the National Cancer Institute (NCI), Cairo University, Egypt has authorized all human subject protocols and procedures (IRB No.: 00004025; approved 20 December 2016).

The clinical and pathological features of the studied groups including age, gender histological type and grade, recurrence and metastasis were collected from the clinical records at the National Cancer Institute (NCI), and they are summarized in Supplementary Table S1. The colitis group in this study was used as a control, since the examinees did not show any histo-pathological changes, only very minimal inflammation in the colonoscopic examination.

### 2.2. Target Panel Design

Our laboratory uses the Ion Torrent (Thermo Fischer Scientific Inc., Waltham, MA, USA) sequencing technologies. The first version of the ready-to-use colorectal cancer gene panel kit based on this technology was the Ion AmpliSeq Colon/Lung panel. This panel appeared a few years ago and was composed of 22 genes. The recent version of this panel from Thermo Fischer is the Ion Torrent Oncomine Colorectal and Pancreatic Panel, composed of 24 genes (Catalog Number: A35121). For our study, we wanted to have a disease focused panel, but with more genes specific to colorectal cancer. To this end, we studied the gene lists available in the commercial cancer gene panels, such as the Comprehensive Cancer Panel (Catalog Number: 4477685) composed of 409 genes, the different versions of the Oncomine Comprehensive panels (161 genes, Catalog Numbers: A33634, A33635, A33757, A33758), and the Qiagen’s GeneRead colorectal panel, composed of 38 genes. This is in addition to studying gene lists from literature, TCGA, and different commercial tests registered in the Gene Test Registry. Our final list included 96 genes, and it is given in Supplementary Table S2. On the one hand, our panel is more comprehensive than the 24 ready-to-use panels. On the other hand, it is still smaller than the CCP and the Oncomine panels, which allows more samples to be sequenced at higher depth and lower cost.

### 2.3. DNA Extraction

The DNA was isolated from the collected biopsies using the QIAamp^®^ DNA mini kit (Cat. No. 51304, Qiagen, Hilden, NRW, Germany) according to the manufacturer’s instructions. The purified DNA was measured using Qubit^®^ 3.0 Fluorometer (Cat. No, Q33216, Thermo Fischer Scientific Inc., Waltham, MA, USA) with Qubit™ dsDNA HS assay kit (Cat. No. Q32854, Thermo Fischer Scientific Inc., Waltham, MA, USA).

### 2.4. Library Preparation and Sequencing

The QIAseq Targeted DNA technology from Qiagen was used to develop kits for our customized gene panel composed of the selected 96 genes (Cat. No. EDHS-10082-002Z-3002 and CDHS-12403Z-675 Qiagen, Hilden, NRW, Germany). The NGS libraries were constructed according to the manufacturer’s instructions. After library preparation, the QIAxcel (Cat No. 900194 Qiagen, Hilden, NRW, Germany) was used to check the fragment size and concentration with the QIAxcel DNA high resolution kit (Cat No. 929002, Qiagen, Hilden, NRW, Germany). The prepared libraries were quantified using QIAseq Library Quant Assay Kit (Cat No. 333304, Qiagen, Hilden, NRW, Germany). Then, the libraries with different sample indexes were combined in equimolar amounts to achieve a similar sequencing depth for each combined library. The fragment size distribution in our libraries ranged between 200 and 1000 bp. The Ion PI Hi-Q Chef Kit (Cat. No. A27198, Thermo Fischer Scientific Inc., Waltham, MA, USA), running on the Ion Chef, was used to load the combined libraries on the Ion PI Chip (Cat. No. A26770, Thermo Fischer Scientific Inc., Waltham, MA, USA). The Ion Proton Platform was used for next generation sequencing using the Ion Proton Sequencing 200 Kit v2 (Cat. No. 4485149, Thermo Fischer Scientific Inc., Waltham, MA, USA).

### 2.5. Bioinformatics Analysis

The Ion Torrent Suite was used for base calling, alignment, and variant analysis. First, the low-quality reads were excluded and the bases with base quality less than Q30 were trimmed. The alignment and variant calling proceeded in two parallel directions to identify the somatic variants: In the first direction, we used the somatic workflow in the Torrent Suite (based on tmap and Torrent Suite Variant Caller) to call the somatic variants. In the second, we used the QIAGEN GeneGlobe Data Analysis Center. Then, the variant lists from the two platforms were combined together and submitted to the annotation workflow. For annotation, we used QIAGEN GeneGlobe Data Analysis Center, Ingenuity Variant Analysis (IVA; QIAGEN) [8], and the Annovar package including COSMIC and population databases. For pathway analysis, we used webgestalt (http://www.webgestalt.org (accessed on 3 November 2021)), Ingenuity Variant/Pathway Analysis (IVA/IPA; QIAGEN), and web Reactome (https:/reactome.org (accessed on 15 November 2021)). For the alignment step, we used the human reference genome version hg19. The sequencing of a sample was accepted if the depth was larger than 100× and the coverage was more than 95% of the target regions.

Somatic mutations in the cancer patients were identified based on the following multi-step filtering process introduced in [9]. First, the variants of low depth (<200×) and quality (<100) were filtered out. Second, non-exonic, non-splicing, and synonymous variants were filtered out. Variants that do not exist in the COSMIC database but that are in population databases with MAF larger than 1% were excluded. Other variants that are not in COSMIC and are classified as benign in Clinvar or HGMD were also excluded. Functional consequences of the identified variants were predicted using Sift [10,11], PolyPhen-2 [12], and CADD [13] tools. To further assess that the remaining variants are somatic, we combined the variants of the control samples (the colitis group) in one database specifying germline mutations, representing a kind of pooled normal variant set. We then used this set of variants to filter out candidate somatic variants that escaped the filtration layers specified above. In other words, known somatic mutations as indicated by COSMIC, provided that they have not been reported before as benign in Clinvar or HGMD, are retained and also the novel candidate somatic variants are retained if their frequency is less than 1% in public database, not in our normal pooled set, and are predicted to affect the protein function. The final list of somatic variants was then reported in tabular format and submitted to the pathway analysis tools IPA and Reactome. Data visualization was performed using R package (version 3.6). The oncoplot and the lollipop plots were visualized using Mutation Annotation Format tools (maftools), R/Bioconductor package [14].

### 2.6. Statistical Analysis

The clinic-pathological features of the assessed patients were analyzed using SPSS software package (version 22). Continuous variables were expressed as mean ± SD and range, while categorical variables were expressed as percentages. Comparisons between groups were analyzed by χ^2^ test or Fisher’s exact test, when appropriate for the categorical variables, and by Mann–Whitney test or Student’s *t*-test when appropriate for the continuous variables. *p*-value was considered significant when *p*-value ≤ 0.05.

## 3. Results

### 3.1. Clinical Features

The patients were classified according to age, gender, histological type, grade, recurrence, and metastasis (Table S1). There were no significant differences in the mean age and gender between the studied groups. The colon was the most affected site in the CP, IBD, and colitis groups, while the rectum was the most affected site reported in 54% of the CRC patients. Regarding the histological features, the adenocarcinoma was the most predominant subtype reported in 82% of the CRC group (*p*-value < 0.001). Nearly half of the CP group (47%) had atypical lesions. The most predominate grade was grade II, found in 64% of the CRC group. Most of the CRC patients presented with non-metastatic and non-recurrent status (98% and 97%, respectively) (Table S1).

### 3.2. The Detected Somatic Mutations in Our Data Set

In total, there were 232 somatic non-synonymous variants (73% SNPs and 27% Indels). The mean depth of coverage of the non-synonymous variants ranged from 500 to 1000× in all studied groups (Figure S1). Most of the variants are in the CRC group, followed by the CP and IBD groups (135 mutations vs. 74 and 23, respectively). The number of cases with at least one somatic mutation was 82 (69%): 45 out of 62 (73%) in the CRC group, 30 out of 38 (79%) in CP, and 8 out of 20 (40%) in the IBD group. That is, the highest diagnostic yield is in the CRC group, followed by the CP and IBD ones.

**Figure 1:**
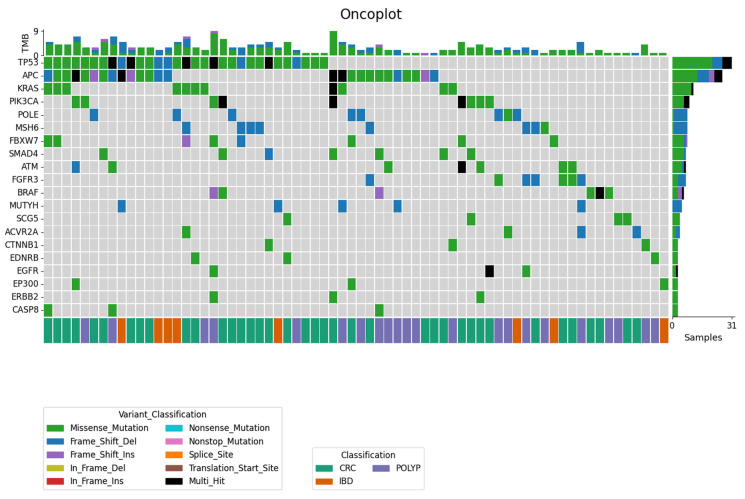
Oncoplot displays the somatic mutations distribution of the top highly mutated genes in different groups. Each column represents a sample, and it is classified according to the group by colors in the last row. Each row represents a particular gene with different variant classification.

Fifty-seven genes out of the studied genes were found to carry somatic mutations (Figure 1). The top genes appearing in all the groups were *TP53* (38%; 31 out of 82 cases carrying at least one somatic mutation), *APC* (32%; 26/82), *KRAS* (13%; 11/82), *PIK3CA* (11%; 9/82), *POLE* (10%; 8/82), *MSH6* (10%; 8/82), *FBXW7* (10%; 8/82), *SMAD4* (9%; 7/81), *ATM* (9%; 7/82), and *FGFR3* (9%; 7/82). In the CRC group, *TP53* (48%; 21 out of 45 CRC cases carrying somatic mutations), *APC* (32%; 14/45), *KRAS* (16%; 7/45), *PIK3CA* (16%; 7/45), *MSH6* (14%; 6/45), *FBXW7* (14%; 6/45), *SMAD4* (14%; 6/45), *ATM* (11%; 5/45), *FGFR3* (9%; 4/45), *BRAF* (7%; 3/45), and *POLE* (7%; 3/45) were the top mutated genes. In the CP group, *APC* (30%; 9 out of 30 CP cases carrying somatic mutations), *TP53* (17%; 5/30), *BRAF* (10%; 3/30), *FGFR3* (10%; 3/30), *KRAS* (10%; 3/30), and *POLE* (10%; 3/30) were the most mutated genes, while *TP53* (62%; 5 out of 8 IBD cases carrying somatic mutations), *APC* (37%; 3/8), and *POLE* (25%; 2/8) were the most mutated ones in the IBD group.

**Figure 2:**
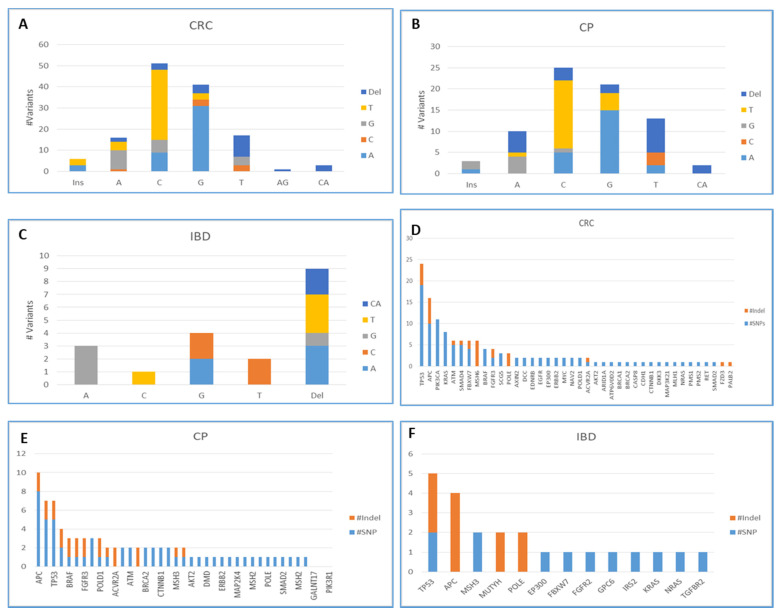
Bar charts (**A**–**C**) show the changes in the reference alleles to the alternative ones in each studied group. Charts (**D**–**F**) show the counts of SNPs and Indels in each mutated gene in each studied group.

As for the somatic mutations that were detected in the top genes in each group, *TP53*, *APC*, and *KRAS* genes harbored the most frequently detected somatic mutations (36, 30, and 12 mutations, respectively) in the total cohort. In the CRC group, *TP53*, *APC*, *PIK3CA*, *KRAS*, and *ATM* genes harbored the most frequently detected somatic mutations (24, 16, 11, 8, and 6 mutations, respectively). In the CP group, *APC*, *TP53*, *BRAF*, *FGFR3*, and *KRAS* genes had the most frequently detected variants (10, 7, 3, 3, and 3). Regarding the IBD group, *TP53* and *APC* genes harbored the most frequent somatic mutations (five and four). The somatic mutational burden per sample in the CRC group was the highest, and it was about three variants per sample on average; this is followed by the CP (2.7) and the IBD groups (2.4). Figure 2 shows the number and the distribution of different SNPs and Indels in each gene in each group. It also shows the number of each type of mutations, including transversion, insertion, or deletion in each group.

**Figure 3:**
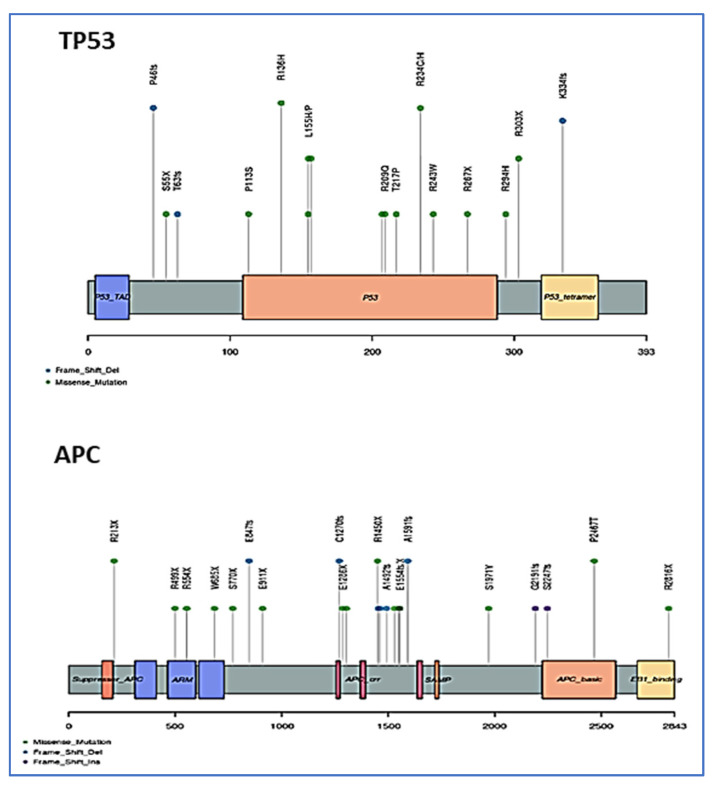
Schematic representation of detected somatic mutations in the TP53 and APC proteins. Frequent mutations in the TP53 at both transactivation and prolin rich regions. Frequent mutations in the APC protein are at the β-catenin binding and down-regulation site. The mutations are colored with respect to their type (missense, frameshift insertion, and frameshift deletions).

As the *TP53* and *APC* genes were found to be the most affected genes, with somatic mutations in all groups, a schematic representation of their somatic mutations at protein level was made, as shown in Figure 3. *TP53* harbored 36 mutations from 31 samples (24 variants from 21 CRC samples, 7 from 5 CP samples, and 5 from 5 IBD) and *APC* harbored 30 mutations from 26 samples (16 variants from 14 CRC samples, 10 from 9 CP, and 4 from 3 IBD). We found that exons 3 and 4 of the *TP53* gene (NM_001126115) possessed a high number of mutations (28% and 25% respectively). In *APC* (NM_001127511), exon 14 harbors most of the mutations (85%). The β-catenin binding and down-regulation site was the most affected region at the APC protein, whereas the transactivation and the proline rich sites were the most affected regions at the TP53 protein.

### 3.3. Common Somatic Mutations Detected in CRC and CP and/or IBD

There are, in total, 16 somatic mutations detected in the CRC group and in either the IBD or the CP group: 10 SNPs and 6 Indels. *TP53* and *APC* are the genes with the greatest number of mutations (five and five mutations, respectively). This is followed by the *KRAS* gene with two mutations, as shown in Table 1. The whole set of the somatic mutations are in the Supplementary Table S3.

### 3.4. Somatic Mutations That Were Identified in CRC Merely and Were Likely Novel in Our Dataset

**Figure 4:**
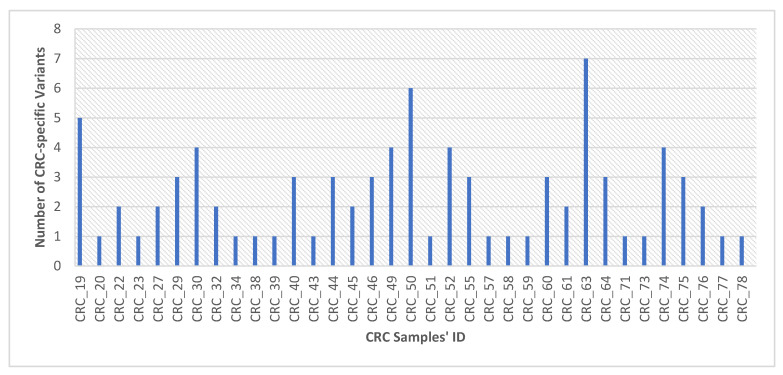
Distribution of somatic variants that were identified merely in CRC samples.

Eighty-four somatic variants harbored by 36 genes were found only in the CRC group, representing 51% of total somatic variants. The top genes carrying CRC-specific mutations are *APC*, *TP53*, *PIK3CA*, *FBXW7*, *ATM*, and *SMAD4*, and they were housing 12, 11, 7, 6, 5, and 5 mutations, respectively. CRC-specific variants harbored by the top genes were reported in 23 out of total 35 CRC patients carrying CRC-specific mutations. Average CRC-specific variants per sample is 2.4, as shown in Figure 4 and Table S3. According to the COSMIC database, we identified 29 somatic variants in 21 genes that were not reported in CRC patients before, as listed in Table 2.

Moreover, ten novel heterozygous mutations were identified, including six in *PIK3CA* (NM_006218) and four in *FBXW7* (NM_001013415), as shown in Table 3. These *PIK3CA* and *FBXW7* mutations were reported in seven and four cases, respectively. Out of these 10 novel mutations, 8 mutations were found in the CRC group only, while a mutation in *PIK3CA* (c.1013T>A) is found in CP group only and a single mutation in *FBXW7* (c.248C>T) is found in IBD group. The ten novel mutations have not been previously reported in any of the public databases.

### 3.5. The Most Commonly Altered Pathways in CRC Patients

**Figure 5:**
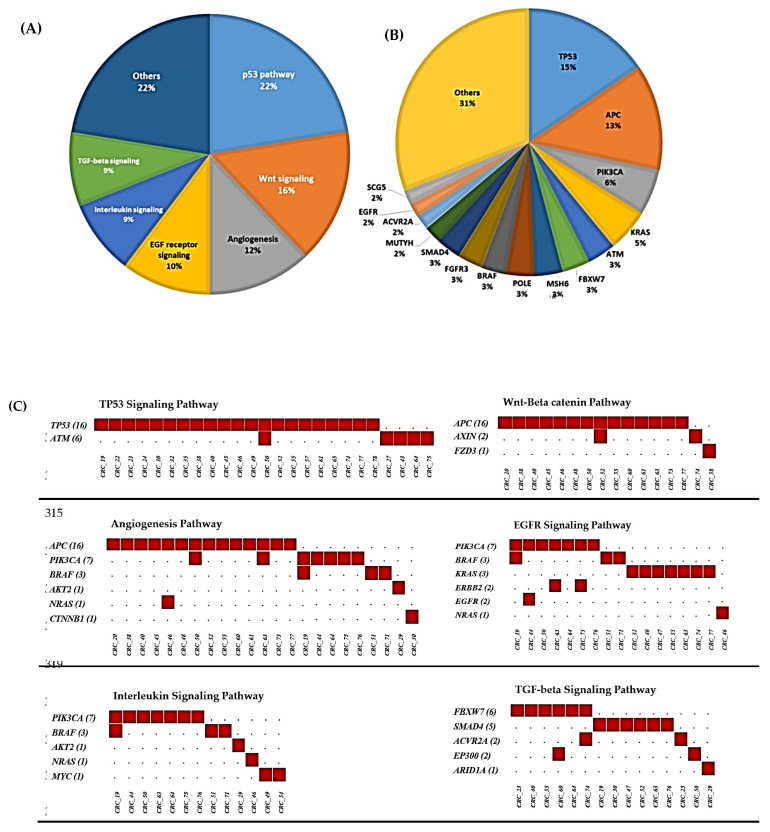
(**A**) Pie chart displaying the most altered pathways in the CRC group. (**B**) Pie chart displaying the distribution of mutations in the cancer driver genes. (**C**) Pathway analysis displays the somatic mutations’ distribution of the genes involved in the P53, Wnt/βcatenin, Angiogenesis, EGFR, Interleukin, and TGF-β signaling pathways; **Number of red squares** indicates the number of identified somatic variants per gene in the CRC patients.

Pathway analysis revealed that the following pathways were strongly suggested to be altered in the CRC group: P53-signaling pathway (*p*-value = 1.28 × 10^−08^), Wnt signaling pathway (*p* = 0.0028), Angiogenesis (*p*-value = 0.00116), EGF-receptor (*p*-value = 0.0012), TGF-beta signaling (*p*-value = 0.0021), and Interleukin signaling (*p*-value = 0.0025). The most altered pathways in CRC patients and the distribution of the mutations of cancer driver genes are in Figure 5.

## 4. Discussion

CRC is one of the leading causes of mortality and morbidity worldwide [1]. To the best of our knowledge, our study is the first to sequence a multiple-gene panel to identify the somatic mutation pattern associated with colon cancer disease progression in a cohort of Egyptian patients to help understand more about colorectal cancer. In the current study, the somatic mutational burden was higher in the CRC patients when compared to the other groups. The *TP53*, *APC*, *PIK3CA*, *KRAS*, and *ATM* were the most frequently mutated genes in the CRC group. Matching with the Cancer Genome Atlas Network, the most frequently altered genes in CRC patients were *TP53* and *APC* [47]. Moreover, it was previously revealed that during cancer initiation, a high mutation level was detected in the *APC* gene. Whereas, elevated mutation levels were observed in *KRAS*, *TP53*, and *SMAD4* during CRC progression [48,49,50].

As for the *TP53*, which is defined as the ‘guardian of the genome’, its alteration is one of the tumor hallmarks and its mutational status is associated with the progression and outcome of sporadic CRC [51]. The *TP53* mutation prevalence rate in Arab CRC patients is 52.5%, while its prevalence rate in their matched Western patients is 47.5% [52]. TP53 was the top-ranked gene in our analysis, as it has been mutated in 38% of the whole CRC cohort. It was the most altered gene with 36 mutations, indicating its role in the transition from an adenoma to carcinoma [53].

Moreover, eleven *TP53* somatic mutations were detected only in the CRC patients and caused loss of functionality. Interestingly, the most affected exons in the *TP53* (NM_001126115) were exon 3 and 4. In accordance with a recent study by Kassem et al. [54] on the Egyptian CRC patients, we found that the four *TP53* somatic mutations c.628C>T, c.448C>T, c.347G>A, and c.128G>A in our cohort are specific to the CRC group, which suggests that they play a key role in the CRC in the Egyptian population. Additionally, we detected two *TP53* variants (c.164C>G and c.137delC) that were previously reported in the esophageal and lung cancers. Interestingly, and according to the COSMIC database, this is the first study to report the presence of such variants in CRC [16,20,21,22,23,24,25,26]. However, further studies are needed to confirm our findings.

Somatic mutations of the *ATM* gene, as a DNA repair gene, occur in many tumor types including colorectal cancer. In colorectal cancer, the loss of ATM protein expression is associated with worse prognosis [55]. Therefore, we are in need of such targeted sequencing studies to help in monitoring the prognosis in Egyptian CRC patients. We have found that the *ATM* gene was mutated in 12% of the CRC cohort; five out of the seven detected somatic mutations were found only in the CRC group. All of the observed ATM mutations had previously been linked to CRC [25], with the exception of two SNPs that were found merely in CRC (c.9007A>G and c.8138G>A). Both of these SNPs have previously been linked to NHL lymphoma [15,16,17,18].

Nowadays, novel therapies have been developed to selectively target patients with ATM-deficient cancers. Those therapies induce synthetic lethality due to lacking an efficient repair mechanism such as platinum drugs [56]. Thus, the *ATM* mutational status could be used to help in the clinical decision-making for those patients along with the development of specific targeted strategies [57]. Thus, it is important to conduct targeted sequencing studies on the Egyptian CRC patients to evaluate the drug efficacy and treatment protocols.

Mutation of the *APC* gene, a multi-functional tumor-suppressor gene, is an early event in the development of CRC and result in activation of Wnt/β-catenin signaling pathway, which is a key event for epithelial development [58]. Mutant *APC*, *Axin2*, and *AMER1* (APC-recruitment protein) disrupt the formation of the β-catenin destruction complex leading to stabilization and accumulation of β-catenin protein, which in turn induces overactivation of Wnt/β-catenin signaling and promotes the proliferation, invasion, and metastasis of cancerous cells [59,60]. We have found that the *APC* gene (NM_001127511) was the second ranked mutated gene (23% of the CRC cohort). There were 12 *APC* somatic mutations with identified loss of function detected only in the CRC group. Interestingly, exon 14 was the most affected exon and it was found to harbor 11 out of 12 detected mutations in the CRC group only. Thus, sequencing this exon could be used as a genetic test assay for CRC diagnosis. Of interest, most of our identified *APC* somatic mutations were located in the β-catenin binding and down-regulation site, which may result in an altered Wnt/βcatenin pathway. Meanwhile, the somatic mutations detected in *AXIN2* (two mutations) and *FZD3* (one mutation) were reported only in CRC patients, and they were participating in Wnt/βcatenin pathway as well. The two *AXIN2* mutations (c.2347G>T and c.1102G>A) were previously addressed as being associated with small cell lung and prostate cancers, respectively [16,30], while the *FZD3* mutation (c.674dupT) was previously reported in oral squamous cell carcinoma (OSCC) [46]. Therefore, this study is the first to report their association with CRC.

The Wnt signaling cascade is mostly activated by *APC* bi-allelic mutations, which in turn decrease the β-catenin degradation [61]. However, *CTNNB1* mutations are considered an alternative method for activation of Wnt signaling pathway [62]. Furthermore, in CRC, the association of *APC* and *CTNNB1* mutations is infrequent [63]. Beta-catenin is encoded by the *CTNNB1* gene. It is another protein that acts as a gene expression regulator for Wnt downstream genes which is responsible for cell proliferation and differentiation [64,65]. Besides regulation of the Wnt signaling pathway, it has a crucial role in cell–cell adhesion through interacting with E-cadherin [66,67,68,69]. In CRC, mutations of *CTNNB1* gene are rare. Here in our study, we identified one *CTNNB1* (NM_001098209) pathogenic variant in CRC patients and it was located on exon 10 (c.1561C>T). This was in agreement with Giannakis et al., who had detected the same mutation in one CRC patient, as well [70].

Epidermal growth factor receptor (EGFR) is a trans-membrane protein. Bad prognosis and drug resistance in CRC is usually associated with EGFR overexpression [71,72]. The available anti-EGFR monoclonal antibodies’ (MoAb) target therapy for metastatic CRC (mCRC) is based on inhibition of the signaling cascade initiated by the binding of EGF to its receptor (EGFR). The mutational status in genes is that being part of the EGFR-signaling pathway (e.g., *KRAS*, *NRAS*, *BRAF* and *PIK3CA*) can determine the response to this target therapy. Hence, they can be used as predictive biomarkers. The following mutations were previously known to contribute to the acquired resistance to anti-EGFR target therapy, mutations in *KRAS* (exons 2, 3, and 4) [73,74,75], *NRAS* (exons 2, 3, and 4), *BRAF* (exon 15), and *PIK3CA* (exon 20) [75,76]. *PIK3CA* and *KRAS* are often co-mutated and therefore can predict the lack of response to anti-EGFR therapy [77,78].

In light of this, the genes implicated in the EGFR-signaling pathway will be discussed in the following lines. *KRAS* and *NRAS* are members of *RAS* gene family. *KRAS* is one of the most frequently mutated genes, with alterations observed in 30–40% of CRC patients. On activation of *KRAS*, a signal transduction cascade will be initiated that will eventually promote many cell processes (e.g., cell differentiation, growth and transformation, apoptosis suppression and angiogenesis) through the subsequent activation of several target effectors (e.g., Raf, Braf, mTOR, MEK1 and 2, ERK, AKT, and PIK3CA) [79,80]. On the other side, *NRAS* mutations are identified in ~4% of CRCs [81]. Additionally, CRC patients with *KRAS* and *NRAS* mutations have less favorable prognoses, shorter survival, and increased tumor aggressiveness [82]. Moreover, *KRAS* and *NRAS* mutations in CRC predict lack of response to anti-EGFR MoAbs therapy [73,83,84]. In this study, the distribution of *KRAS* and *NRAS* mutations among our CRC patients were detected. In CRC patients, three were found to carry *KRAS* mutations in codon 13 (c.38G>A) while five CRCs carried *KRAS* mutations in codon 12 (c.35G>A; c.35G>T) and both codons are located on exon 2. As for the *NRAS* mutations, one mutation in codon 12 on exon 2 (c.35G>T) was identified in one CRC patient only.

Serine or threonine protein kinase is encoded by a proto-oncogene (*BRAF*) which encompasses 18 exons. This protein is associated with the MAPK pathway, which is involved in carcinogenesis of many cancers [85,86]. *BRAF* mutation leads to a constitutive activation of the MAPK signaling pathway, eliciting cellular proliferation, angiogenesis, and differentiation [87]. Up to now, around 30 *BRAF* alterations have been identified [88]. *BRAF* mutations were observed in less than 10% of CRC. *BRAF* mutations may indicate an initial event in tumorigenesis [89,90,91]. The most frequent *BRAF* mutation is the V600E subtype (c.1799T>A; exon 15) [92]. This mutation increases the activity of *BRAF* 10 times relative to the *BRAF* wild type, which in turn promotes cell survival via the ERK or MEK signaling cascade [93]. Our data were in concordance with this, as we identified c.1799T>A in two CRC patients. c.1799T>A is linked to worse prognosis and resistance to standard therapies in CRC (e.g., EGFR inhibitors) and indicates tumor aggressiveness [94]. To date, there were no encouraging findings regarding usage of *BRAFV600E* inhibitors in CRC [90,95]. Most of the non-V600 mutations are scarce in CRC (~2%), and they were associated with better prognosis compared withV600 mutations [96,97]. Here, we have identified two non-*BRAFV600* mutations in CRC patients (c.1781A>G and c.1796C>G; both are on exon 15) and these findings were also in agreement with Yanus et al. and Won et al. [98,99].

*PIK3CA* mutations are frequently associated with other gene mutations which are involved in significant cancer-related pathways, such as the Wnt/beta-catenin pathway and tyrosine kinase receptors K-Ras/BRAF/MAPK. The *PIK3CA* gene encodes the alpha catalytic subunit of phosphatidylinositol-4,5-bisphosphate 3-kinase (PI3K), which is mutated in several malignancies (e.g., breast, ovary, lung, and CRC) [100]. *PIK3CA* mutations have been detected in 10 to 32% of colorectal tumors [101,102]. In our CRC patients, we have detected seven *PIK3CA* somatic variants; out of these, five of them were likely to be novel (c.40C>A, c.42C>G, c.44T>G, c.2992T>C and c.2825A>G), and they were located on exons 2, 2, 2, 21, and 20, respectively. In addition, our results are in agreement with Samuels et al., who has reported an association between *PIK3CA* mutation (c.3140A>G “p.H1047R”, exon 21) and CRC [103]. Our study is the first to show an association between the seventh *PIK3CA* variant (c.3157A>G, exon 21) detected in our CRC patients and colorectal cancer. This variant was previously addressed with lung cancer [38]. Furthermore, our CRC patients had an apparent low frequency of the significant *KRAS*, *BRAF*, and *PIK3CA* mutations, which was consistent with an Egyptian study by Farghal et al. [104].

Matching with what we have mentioned before, we have detected somatic mutations in eleven CRC patients (18%) that are linked to the resistance to anti-EGFR target therapy, three variants in *KRAS* located on exon 2, one mutation in *NRAS* located on exon 2, one *BRAFV600*, two non-*BRAFV600* mutations located on exon 15, and one *PIK3CA* mutation located on exon 20. Thus, these data may provide beneficial information that helps in the clinical management regarding anti-EGFR therapy using a personalized medicine approach for the colorectal cancer patients in Egypt. In addition, we detected other mutations in CRC patients that are known to participate in the EGFR-signaling pathway, such as *ERBB2* (c.922G>A, c.2690G>A), and *EGFR* (c.2116C>T and c.630G>A).

When we performed pathway analysis, it revealed dysregulations of six pathways. Inactivation of P53 signaling pathway was detected in CRC patients due to the presence of deleterious mutations in *TP53* and *ATM* genes. Up-regulation of the Wnt/βcatenin pathway in CRC patients due to mutations in *APC*, *AXIN2*, and *FZD3* reveal that the Wnt/βcatenin pathway plays a major role in sporadic colorectal carcinogenesis. Therefore, the dysregulation of both pathways in our CRC group may arise as an attractive therapeutic target [105,106]. Meanwhile, high levels of angiogenesis are one of the most known clinical features in CRC, as cancer cells are reliant on neovascularization for oxygen and nutrients for enhancement of their survival and progression [107]. On activation of Wnt/β-catenin signaling pathway, the expression of Wnt/β-catenin downstream genes involved in angiogenesis are surged up due to the high levels of nuclear β-catenin [108]. In addition, mutations in the *RAS* gene can lead to PI3K/AKT/mTOR pathway activation that will promote the expression of other angiogenesis-associated factors (e.g., VEGF, nitric oxide, and angiopoietins) [109]. Angiogenesis is also mediated by the MAPK signaling pathway via BRAF (serine/threonine protein kinase) [110]. Moreover, tumor angiogenesis can also be promoted by activation of endothelial nitric oxide through *AKT* mutations [111]. In the present study, considering the identified mutations in *APC*, *CTNNB1*, *NRAS*, *PIK3CA*, *AKT2*, and *BRAF* genes in our CRC patients, the dysregulation of the Angiogenesis pathway could be attributed to the interaction between Wnt/β-catenin, PI3K/AKT/mTOR, and MAPK signaling pathways, and this is in agreement with Lee et al. and Jeong et al. [48,112,113]. Thus, the use of anti-angiogenic therapy will be of great benefit, as it can inhibit both cancer cell growth and dissemination [108].

Matching with two studies that reported the association of *SMAD4* mutations with the CRC, we detected five somatic mutations only in the CRC group [25]. The *SMAD4* gene acts as an intracellular mediator of TGF-β superfamily signals. TGF-β/SMAD4 signaling maintains DNA damage response (DDR) and DNA damage repair [114]. Additionally, it acts as anti-angiogenic by interacting with the Wnt signaling pathway [115]. In this study, the TGF beta pathway was down-regulated in the CRC patients. It was suggested that loss or down-regulation of the *SMAD4* promotes malignant progression via acquiring resistance to TGF-β superfamily growth inhibition [116]. Moreover, its loss shifts the TGF-β signaling pathway to a tumor promoter instead of a tumor suppressor [117]. Isaksson-Mettavainio et al. reported that loss of the *SMAD4* occurs in the CRC in frequencies ranging from 9 to 67% [118]. Moreover, the *SMAD4* loss was also associated with worse clinical outcome and resistance to fluoropyrimidine-based chemotherapy [119], implicating its use as a prognostic marker in the CRC patients [120]. Thus, we propose that the Egyptian CRC patients carrying *SMAD4* mutations may not benefit from fluoropyrimidine-based treatment.

The *EP300* gene has been previously observed in gastric, breast, pancreatic, and colorectal cancers. In addition, Gayther et al. reported a great relevance of the *EP300* loss in colorectal carcinogenesis [121]. Our study found that the *EP300* gene harbored two missense mutations in CRC patients (c.1058G>A and c.3671+1G>A). They were previously detected in breast and gastric cancers, respectively [31,35]. Moreover, Huh et al. reported that *EP300* overexpression was an indicator of good prognosis in the CRC patients [122]. Therefore, the identified somatic mutations in the *EP300* gene might serve as a predictor of bad prognosis in Egyptian CRC patients. One of the most frequently detected somatic mutations in the CRC is in the tumor suppressor *FBXW7* gene. Loss of the *FBXW7* was reported to promote epithelial–mesenchymal transition (EMT) and metastasis in the CRC cells [123]. The present study reported six somatic mutations in the *FBXW7* gene that were found associated with CRC: two were previously reported with CRC patients (c.1647delG, c.823C>T), a single mutation was previously reported with breast cancer but was not addressed with CRC before (c.167A>G), and another three likely novel mutations (c.1568C>A, c.1252dupA, c.1030T>C) [16,25,124].

The functional loss of the tumor suppressor *ARIDA1* gene has been previously reported as a frequent event in the colorectal carcinogenesis [125,126]. However, our study showed a low frequency of *ARIDA1* mutations in CRC patients (only a single case in the CRC group). *ACVR2A* (activin A receptor type 2A) has a mutation rate of about 60%, making it the most frequently mutated gene in hypermutated colon cancer [47]. It mediates the actions of activins, which are ligands belong to TGF- β family with diverse biological functions [127]. Here, we detected two mutations (missense mutation; c.217A>G and Frameshift del mutation; c.303delT) in CRC patients.

Regarding the common somatic mutations detected in all the studied groups, we have found that four mutations were the most frequent (c.137delC and c.422G>A in *TP53*), (c.38G>A in *KRAS*), and (c.4337_4338del in *POLE*). We found no significant increase in frequency of those mutations from IBD to, finally, CRC. Thus, we recommend increasing sample size to validate the association of these variants with disease progression in further studies.

## 5. Conclusions

To the best of our knowledge, this is the first study on the sequencing of a multiple-gene panel for disease progression of Egyptian CRC patients. Our results revealed that *APC*, *TP53*, *PIK3CA*, *FBXW7*, *ATM*, and *SMAD4* were the top genes carrying CRC-specific mutations. *APC* and *TP53* genes were the most affected genes in all groups; most of their deleterious somatic mutations were located in exon 14 and exon 3, respectively. Twenty-nine somatic mutations in 21 genes were found to be associated with CRC patients exclusively. Additionally, ten likely novel variants in *PIK3CA* and *FBXW7* were identified in Egyptian CRC patients. P53, Wnt/βcatenin, Angiogenesis, EGFR, TGF-β, and Interleukin signaling pathways were found to be the most altered pathways in CRC patients. Furthermore, our findings revealed that 18% of CRC patients had somatic mutations linked to resistance to anti-EGFR target therapy, implying that 82% of patients could benefit from this treatment. This study may provide a better understanding of colorectal cancer and identification of cancer driver genes with cancer-specific variants in our patients, and these findings may assist in the development of diagnosis and novel personalized treatment regimens suited to Egyptian colorectal cancer patients. It is worth noting that our findings are confined to grade II patients because they account for 64% of all CRC cases.

## Figures and Tables

**Table 1:** Highly frequent somatic mutations detected in the CRC group and in the CP or the IBD group.

Gene	Position	ID	Type	Class	HGVS.c	HGVS.p	IBD (*n* = 20)	CP (*n* = 38)	CRC (*n* = 62)
*TP53*	chr17:7579433	COSM6970737	INDEL	PV	c.137delC	p.P46fs	1	2	2
	chr17:7572991	COSM6806501	INDEL	VUS	c.722delA	p.K241fs	2	0	2
	chr17:7577121	COSM99933	SNP	CIP/PV ^#^	c.421C>T	p.R141C	0	1	3
	chr17:7577120	COSM1645335	SNP	PV-LPV/PV ^#^	c.422G>A	p.R141H	1	1	1
	chr17:7578263	COSM99666	SNP	PV/PV ^#^	c.190C>T	p.R64X	0	1	1
*APC*	chr5:112116592	COSM13134	SNP	PV/PV ^#^	c.667C>T	p.R223X	1	0	1
	chr5:112173831	COSM201301	INDEL	PV	c.2486delA	p.E829fs	1	0	1
	chr5:112175101	COSM19262	INDEL	PV	c.3756delT	p.C1252fs	1	1	0
	chr5:112175639	COSM13127	SNP	PV/PV ^#^	c.4294C>T	p.R1432X	0	1	1
	chr5:112178690	COSM4169178	SNP	CIP/PV ^#^	c.7345C>A	p.P2449T	0	1	1
*ACVR2A*	chr2:148657066	COSM5192837	INDEL	VUS	c.303delT	p.Y101fs	0	1	1
*KRAS*	chr12:25398281	COSM532	SNP	PV/PV ^#^	c.38G>A	p.G13D	1	1	3
	chr12:25398284	COSM1135366	SNP	PV/PV ^#^	c.35G>A	p.G12D	0	1	3
*BRAF*	chr7:140453136	COSM476 *	SNP	PV/PV ^#^	c.1799T>A	p.V600E	1	0	2
*MSH6*	chr2:48030692	COSM6715812	INDEL	PV	c.2916delT	p.T972fs	0	1	6
*POLE*	chr12:133220099	COSM1745059	INDEL	VUS	c.4337_4338del	p.V1446fs	2	2	3

**HGVS.c:** Human Genome Variation Society, Coding DNA sequence; **HGVS.p:** Human Genome Variation Society, protein sequence; **Chr.**: Chromosome. **IBD**: Inflammatory Bowel Disease; **CP**: Colonic Polyp; **CRC**: Colorectal Cancer. Variants were Classified for their pathogenicity according to ClinVar and FATHMM (**^#^**) predictions; **PV**: Pathogenic Variants; **LPV**: Likely Pathogenic Variant; **VUS**: Variants of Uncertain Significance; **CIP**: Conflicting Interpretation of Pathogenicity; **N**: Neutral. (*) **indicates a Drug Response Variant.**

**Table 2:** The identified somatic variants that were not previously addressed in colorectal cancer.

Gene	Position	Exon	ID	Type	Class	HGVS.c	HGVS.p	CRC Cases	Previously Reported in Cancers of:	References
*ATM*	chr11:108205823 *	55	COSM21829	SNP	VUS/PV ^#^	c.8138G>A	p.R2713K	1	Haematopoietic and lymphoid, Urinary tract	[15,16]
	chr11:108236071 *	63	COSM3733315	SNP	PV ^#^	c.9007A>G	p.N3003D	1	Endometrium, Haematopoietic and lymphoid	[17,18]
	chr11:108181014	39	COSM2110552	SNP	CIP/PV ^#^	c.5890A>G	p.K1964E	2	Haematopoietic and lymphoid	[19]
*TP53*	chr17:7579406 *	3	COSM2745056	SNP	PV ^#^	c.164C>G	p.S55X	1	Oesophagus, Haematopoietic and lymphoid, Lung, Billiary tract, Urinary tract	[16,20,21,22,23]
	chr17:7579433	3	COSM6970737	INDEL	PV	c.137delC	p.P46fs	2	Endometrium, Lung, Billiary tract, Stomach	[24,25,26,27]
*SCG5*	chr15:32935813 *	2	COSM700179	SNP	N ^#^	c.20C>G	p.S7C	1	Lung	[16]
	chr15:32983953	5	COSM4607013	SNP	VUS/PV ^#^	c.532C>T	p.R178X	2	Adrenal gland, Haematopoietic and lymphoid	[28,29]
*AXIN2*	chr17:63530088 *	10	COSM317040	SNP	VUS/PV ^#^	c.2347G>T	p.A783S	1	Lung	[30]
	chr17:63534419 *	5	COSM6979947	SNP	VUS/PV ^#^	c.1102G>A	p.A368T	1	Endometrium, Prostate	[16,25]
*EP300*	chr22: 41523642 *	4	COSM6566095	SNP	PV ^#^	c.1058G>A	p.R353H	1	Breast	[31]
	chr22:41556727 *	20	COSM84765	SNP	LPV/PV ^#^	c.3671+1G>A	p:NA	1	Osephagus, Thyroid, Breast	[32,33,34]
*FGFR3*	chr4:1806083 *	9	COSM4748566	SNP	PV ^#^	c.1102G>A	p.E368K	1	Stomach	[35]
	chr4:1806220 *	9	COSM4604190	SNP	PV ^#^	c.1239G>C	p.K413N	1	Upperaerodigestive tract	[36]
*NAV2*	chr11:20101669 *	15	COSM3383386	SNP	PV ^#^	c.2431G>A	p.V811I	1	Stomach, Pancrease	[16,37]
	chr11:20122686 *	22	COSM2112039	SNP	PV ^#^	c.3577G>A	p.A1193T	1	Stomach, Endometrium	[16]
*PIK3CA*	chr3:178952102 *	21	COSM3724544	SNP	PV ^#^	c.3157A>G	p.T1053A	1	Lung	[38]
*FBXW7*	chr4:153271257 *	2	COSM7344083	SNP	PV ^#^	c.167A>G	p.H56R	1	Breast	[39]
*EGFR*	chr7:55268077 *	18	COSM7327079	SNP	VUS/PV ^#^	c.2116C>T	p.R706X	1	Prostate	[16]
*AKT2*	chr19:40741933 *	9	COSM5855773	SNP	PV ^#^	c.910C>T	p.R304C	1	Melanoma	[40]
*ARID1A*	chr1:27092791 *	9	COSM5992207	SNP	VUS/PV ^#^	c.2812G>A	p.A938T	1	Prostate	[41]
*BRCA1*	chr17:41228557 *	12	COSM6943771	SNP	N ^#^	c.4291G>C	p.E1431Q	1	Urinary tract	[25]
*BRCA2*	chr13:32972525 *	27	COSM4990374	SNP	CIP/PV ^#^	c.9875C>T	p.P3292L	1	Ovary, Skin, Prostate	[42,43,44]
*DCC*	chr18:50936877 *	20	COSM4072554	SNP	PV ^#^	c.2991G>A	p.M997I	2	Skin, Stomach	[16,45]
*FZD3*	chr8:28384945 *	4	COSM5979515	INDEL	VUS ^#^	c.674dupT	p.T225fs	1	Upper aerodigestive tract	[46]
*MYC*	chr8:128748843 *	1	COSM6206407	SNP	PV ^#^	c.4G>A	p.D2N	2	Haematopoietic and Lymphoid	[16]
*PMS1*	ch2:190670454 *	3	COSM6938193	SNP	PV ^#^	c.209C>T	p.S70F	1	Prostate	[25]
*PMS2*	chr7:6029533 *	8	COSM6923151	SNP	VUS/PV ^#^	c.724G>A	p.E242K	1	Breast	[25]
*ACVR2A*	chr2:148657066	3	COSM5192837	INDEL	VUS	c.303delT	p.Y101fs	2	Breast	[16]
*CASP8*	chr2:202134265	4	COSM7339941	SNP	N ^#^	c.338C>A	p.A113E	2	Thyroid	[34]

**HGVS.c**: Human Genome Variation Society, Coding DNA sequence; **HGVS.p**: Human Genome Variation Society, protein sequence; **Chr.:** Chromosome. **IBD**: Inflammatory Bowel Disease; **CP**: Colonic Polyp; **CRC**: Colorectal Cancer. Variants were Classified for their pathogenicity according to ClinVar and FATHMM (**^#^**) predictions; **PV**: Pathogenic Variants; **LPV**: Likely Pathogenic Variant; **VUS**: Variants of Uncertain Significance; **CIP**: Conflicting Interpretation of Pathogenicity; **N**: Neutral. (*) **indicates Variants Appeared in CRC Only.**

**Table 3:** Likely Novel Mutations detected in the cohort.

Gene	Position	Exon	ID	Type	HGVS.c	HGVS.p	Occurrence
*PIK3CA*	chr3:178916657	2	.	SNP	c.44T>G	p.L15W	CRC = 3
	chr3:178916653	2	.	SNP	c.40C>A	p.H14N	CRC = 2
	chr3:178916655	2	.	SNP	c.42C>G	p.H14Q	CRC = 2
	chr3:178921531	5	.	SNP	c.1013T>A	p.I338N	CP = 1
	chr3:178948053	20	.	SNP	c.2825A>G	p.K942R	CRC = 1
	chr3:178951937	21	.	SNP	c.2992T>C	p.F998L	CRC = 1
*FBXW7*	chr4:153244235	11	.	SNP	c.1568C>A	p.S523X	CRC = 1
	chr4:153247195	9	.	INDEL	c.1252dupA	p.T418fs	CRC = 1
	chr4:153249394	8	.	SNP	c.1030T>C	p.S344P	CRC = 1
	chr4:153268206	3	.	SNP	c.248C>T	p.P83L	IBD = 1

**HGVS.c**: Human Genome Variation Society, Coding DNA sequence; **HGVS.p**: Human Genome Variation Society, protein sequence; **Chr.:** Chromosome; **IBD**: Inflammatory Bowel Disease; **CP**: Colonic Polyp; **CRC**: Colorectal Cancer.

## Data Availability

All data generated or analyzed during this study and its supplementary information files are included in this published article.

## Supplementary Materials

The following are available online at https://www.mdpi.com/article/10.3390/cimb44030090/s1, Figure S1: (A) Coverage depth of the variants in the studied groups, Table S1: Clinicopathological features of the studied groups, Table S2: The customized gene list. Table S3: The somatic mutations detected in the studied groups.
