# Supplementary material for: Multigene Panel Sequencing Reveals Cancer-Specific and Common Somatic Mutations in Colorectal Cancer Patients: An Egyptian Experience
Source: Curr Issues Mol Biol. 2022 Mar 18;44(3):1332–52. doi: 10.3390/cimb44030090 (PMC8947625; doi:10.3390/cimb44030090)
Supplement: Supplementary file 1 [file cimb-44-00090-s001.zip › Tables S1 and S2.pdf]

Table S1: Clinicopathological features of the studied groups.

	CRC<br>n= 62	Polyp<br>n=38	IBD<br>n=20	Control<br>n= 20	*P - value*
**Age:**					
**Mean ± SD**	51±12a	54±12a	45±15a	51±16a	0.151
**(Range)**	(24-76)	(26-74)	(22-74)	(21-77)	
**Gender**					
**Male**	28 (45%) a	20 (53%) a	10 (50%) a	10 (50%) a	0.59
**Female**	34 (55%) a	18 (47%) a	10 (50%) a	10 (50%) a	
**Histological type**	Adenocarcinoma (82%) [a]<br>Mucinous adenocarcinoma (6%) [b]<br>Signet Ring adenocarcinoma (5%) [b]<br>Neuroendocrine carcinoma (5%) [b]<br>Squamous cell carcinoma (2%) [b]	Typical lesion (40%) [a]<br>Atypical lesion (47%) [a]<br>Mixed lesions (13%) [a]	-	-	<0.001
**Site**					
**Colon**	20 (33%) [a]	30 (79%) [a]	11 (55%) [a]	16 (80%) [a]	<0.001
**Rectum**	34 (54%) [a]	6 (16%) [b]	9 (45%) [a]	2 (10%) [b]	
**Sigmoid**	8 (13%) [a]	2 (5%) [b]	0 (0%) [b]	2 (10%) [b]	

**Grade**					
**In situ**	1 (2%) [b]				
**II**	40 (64%) [a]	-	-	-	<0.001
**III**	3 (5%) [a]				
**NA**	18 (29%) [a]				
**Metastasis**		-	-	-	
**Yes**	1 (2%) [a]				<0.001
**No**	61 (98%) [b]				
**Recurrence**		-	-	-	<0.001
**Yes**	2 (3%) [a]				
**No**	60 (97%) [b]				

Table S2: The 96 customized gene list.

EDHS-10082-002Z-3002 (70 genes) & CDHS-12403Z-675 (26 genes)

ACVR1B	BRCA1	DCC	FGFR3	KLF5	MYC	PTEN	SOX9
ACVR2A	BRCA2	DKK	FHIT	KRAS	MYO1B	PTPN11	SRC
AKT1	BUB1B	DMD	FLCN	MAP2K4(MKK4)	NAV2	PTPN12	STK11
APC	CASP8(FLICE)	EDNRB	FZD3	MAP7	NRAS	RBFOX1	TCERG1
ARIDIA	CD27	EGFR(ERBB1)	GALNT12	MET	PALB2	RET	TCF7L1
ATM	CDH1(E-Cadherin)	ENG(EVI-1)	GPC6	MIER3	PARK2	SCG5	TCF7L2
ATP6V0D2	CDK4	EP300	GREM1	MLH1	PIK3CA	RPS20	TGFBR2
AXIN2	CDK8	EPCAM	HFN4A	MLH3	PIK3R1	SCN5A	TP53
BAX	CDKN2A(p161nk4a)	ERBB2(HER-2)	IGF2	MSH2	PMS1	SLC9A9	TTN
BLM	CHECK2(RAD53)	FAM123B(WTX)	IRS2	MSH3	PMS2	SMAD2	USP12
BMRP1A (ALK3)	CTNNA1	FBWX7	KIAA1804	MSH6	POLD1	SMAD3	VT11A
BRAF	CTNNB1	FGFR2	KIT	MUTYH	POLE	SMAD4	WBSCR17
